# Community Health Assessment Tools Adoptable in Nursing Practice: A Scoping Review

**DOI:** 10.3390/ijerph20031667

**Published:** 2023-01-17

**Authors:** Chiara Pazzaglia, Claudia Camedda, Nikita Valentina Ugenti, Andrea Trentin, Sandra Scalorbi, Yari Longobucco

**Affiliations:** 1 Bologna Local Health Trust, 40124 Bologna, Italy; 2 IRCCS Azienda Ospedaliero-Universitaria di Bologna, 40138 Bologna, Italy; 3 Attorney at Law, 40138 Bologna, Italy; 4 Department of Medical and Surgical Sciences, University of Bologna, 40126 Bologna, Italy; 5 Department of Health Sciences, University of Florence, 50139 Florence, Italy

**Keywords:** family and community nursing, COVID-19, community health assessment, primary healthcare, health promotion

## Abstract

The WHO European Region defined the role of a new central professional for primary care, the Family and Community Nurse (FCN). The introduction of an FCN in the framework of health policies highlights a key role of nurses in addressing the needs of families and communities. A scoping review was conducted in order to identify and describe the available tools which have been adopted for the assessment of community health needs by FCNs. A comprehensive literature review on the Embase, Cochrane Library, PubMed, CINAHL, Scopus and PsycInfo databases was conducted including all studies up to May 2021. A total of 1563 studies were identified and 36 of them were included. The literature review made it possible to identify studies employing twelve different community assessment tools or modalities. Referring to the WHO framework proposed in 2001, some common themes have been identified with an uneven distribution, such as profiling the population, deciding on priorities for action and public healthcare programs, implementing the planned activities, an evaluation of the health outcomes, multidisciplinary activity, flexibility and involving the community. To the best of our knowledge, this work is the first attempt to provide an overview of community assessment tools, keeping the guidance provided by the WHO as a reference.

## 1. Introduction

During 2020, the whole world had to face, and is still fighting, the consequences of the pandemic caused by COVID-19, which led to an unprecedented health crisis, not only due to the organic shortages of health professionals, procurement materials and personal protective equipment, but also due to the organization of Western health systems that are built around the concept of patient-centered care. The concept of patient-centered care does not allow for addressing the changes that an epidemic requires as an approach based on community-centered care [1].

These two care models do not express alternative concepts, but rather a cross planning and organizational approach; therefore, these are complementary concepts. Ideally, the patient-centered model responds more to the individualistic–paternalistic ethics, while the community-centered model responds to the ethics of public assistance systems, oriented to the fairest possible effort to meet the needs of the entire population, therefore of each person. This second model integrates more easily with the possible decision-making and organizational synergies between health, society and the environment.

The WHO has identified the development of community health systems as a health policy framework goal [2]. Primary healthcare (PHC) has been identified, starting from the 1978 Alma-Ata Declaration, as an integral part of the health system of each country that inextricably links health to the whole social and economic development of the community, based on equity, community participation, prevention, appropriate technology and intersectoral and integrated approaches to development [3]. Policies must ensure that activities and processes referring to the population derive from a careful assessment of local socio-health needs; evidence-based approaches must be applied to understand the inequalities in community health. The identification of a population’s unmet health needs, and the changes needed to meet them, are crucial elements for health professionals seeking to plan appropriate and effective programs to improve or initiate new services [4].

According to another approach, nursing care is carried out on the basis of how individuals, families and communities are conceptualized and of how nurses collaboratively work with them, taking into account organizational values and beliefs [5]. People are always members of their own families and communities, even when they are analyzed and assisted individually [6]. The health of individuals, families and communities influence each other [6]. The WHO [7] defined community by referring to its members, while respecting their group dimension and in relation to their specific identity connotations. In Italy, the nursing figure and professional profile are outlined in the Ministerial Decree n. 739, 14 September 1994. Article 1, paragraph 3, in particular, provides the following: “3. The nurse: (a) participates in the identification of persons’ and community’s health needs; (b) identifies the nursing care needs of individuals and community and formulates the related objectives; (c) plans, manages and evaluates the nursing intervention;…. (d) acts both individually and in collaboration with other health and social professionals”. The interpretation of the Decree lets emerge, first, the distinction between “individual and community health needs “ and “individual and community nursing care needs”. This difference, therefore, allows us to state that—with regards to the health needs—nurses act together with other professionals while—with regards to the nursing care needs—nurses are assigned an exclusive competence. Furthermore, from the Decree’s interpretation emerges that nurses “(c) plan, manage and evaluate the nursing care intervention” but not the public health programs and services.

The WHO European Region [8] defined the role of a new central professional for primary care, the Family and Community Nurse (FCN). The introduction of an FCN in the framework of health policies highlights a key role of nurses in addressing the needs of families and communities. These needs can affect the whole course of people’s lives from health to illness, with reference to the needs of the most vulnerable social groups, through a comprehensive understanding of the determinants of health, primary healthcare and public health principles. Community nursing therefore leads to a community care process, the activation of formal, informal and technological networks, enhances health professionals’ own area of competences and implements an approach aimed at enhancing individuals’, families’ and communities (relatives, friends, neighbors, volunteer groups, self-help, etc.) resources.

Therefore, through a comprehensive evaluation process which is as thorough and in-depth as possible for the context, nurses need to establish a broad knowledge of the community and its needs. This assessment is carried out regularly through a continuous process that allows the planning of not only the interventions but also the public health programs according to the phases described below [9].

− Profiling:
  ○ Collection of relevant information that will inform the nurse about the health state and needs of the population;
  ○ Analysis of this information to identify the major health issues.
− Deciding on priorities for action;
− Planning public health and healthcare programs to address the priority issues;
− Implementing the planned activities;
− Evaluation of the health outcomes.

Family and community assessment involves data collection on what the community needs [9]. The objectives of the assessment process are:

− To identify community strengths and areas for improvement;
− To identify and understand the state of the community’s health needs;
− To define areas for improvement to guide the community towards the implementation and support of policies, systems and environmental changes around healthy living strategies (e.g., physical activity, nutrition, tobacco and chronic disease management);
− To help prioritize community needs and to consider the appropriate allocation of available resources.

The community needs assessment enables local stakeholders to work together in a collaborative process to analyze the community itself; offer suggestions and examples of change policies, systems and strategies; provide feedback to communities as they institute local changes for healthy living [9]; ensure resources allocation where there is the greatest health benefit; and adopting the principle of equity in practice [4].

Increasing healthcare demands, limited resources and growing health inequalities require governments across the European community to guarantee the right to health of all citizens [1], resulting in a paradigm shift away from historic “wait-and-see” healthcare in Europe and towards one already prevalent in other countries [10]. The issue of the assessment of community health needs, or rather socio-health needs, fits into this context, not only in European health services but in those around the world. The “health needs assessment” process plays, in fact, a central role: it allows professionals and policy-makers to identify priority health needs in the population and to ensure that social and health resources are used to maximize health and well-being; however, despite the centrality of this issue, there is not currently a standardized tool which reflects the framework proposed by the WHO in 2001 [9].

A scoping review was conducted in order to identify and describe the available tools which have been adopted for the assessment of community health needs by FCNs, without geographical restrictions.

## 2. Materials and Methods

The scoping review allows researchers to examine the extent and nature of research activities on a specific topic, to summarize and disseminate research findings and to identify research gaps in the existing literature [11].

A comprehensive literature review on the Embase, Cochrane Library, PubMed, CINAHL, Scopus and PsycInfo databases was conducted including all studies up to May 2021 in the English or Italian languages. The following inclusion criteria were adopted:

− Primary and secondary studies, abstract and full text available;
− Community, family and ethnic minorities’ needs assessment process;
− Assessment/measurement tools (in particular validation studies);
− Family and community nursing role;
− Primary care context.

The search terms included were: “family nursing”, “community nursing”, “community health services”, “needs assessment”, “assessment tool”, “assets assessment” and “health needs”. These search terms were combined with each other through the use of Boolean operators and wildcard characters for the different databases, in order to obtain as many results as possible.

Four reviewers screened the title and abstracts and selected the eligible articles. All studies that discussed or applied community assessment tools or models were included.

The full text articles of all potentially eligible studies were retrieved and, after removing the duplicates, reviewed independently by four reviewers (CC, CP, NVU and YL). Any disagreement was resolved by a majority vote with the support of a tiebreaker (SS).

Data of the included studies were extracted and synthetized, in particular: authors, year, title, setting, study design and methods, purpose, sample and adopted tool and main findings. Any disagreement in the data extraction was resolved by a consensus of two experts (YL and SS). The study authors or investigators were contacted when additional information was necessary [12].

## 3. Results

### 3.1. Study Selection and Charting the Data

**Figure 1:**
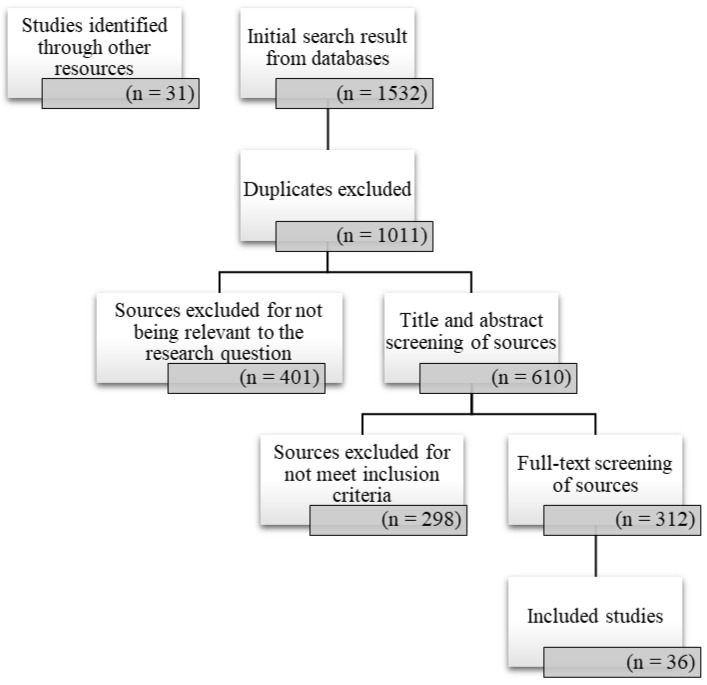
Study selection flow-chart.

After the removal of duplicates, articles were screened in order by titles, abstracts and then full text. A total of 1563 studies were identified and, after the removal of the duplicate studies, 610 abstract and 312 free-full-text studies were evaluated and then a total of 36 studies were identified (Figure 1).

### 3.2. Data Extraction

A total of 32 of the included studies are primary studies—USA (n = 16), UK (n = 3), Canada (n = 2), Japan (n = 2), Australia (n = 1), China (n = 1), Honduras (n = 1), Portugal (n = 1), Singapore (n = 1), South Korea (n = 1), Sweden (n = 1), Vietnam (n = 1)—and 4 are secondary studies.

The characteristics of primary studies are heterogeneous, also in the adopted design.

A synthesis of the results is reported in Table 1.

The most widespread tool, although only officially recognized in the USA, is the Community Health Needs Assessment (CHNA), reported by Akintobi et al. [13]; Carlton and Singh [14]; Cain et al. [15]; Evans-Agnew et al. [16]; Pennel et al. [17]; Pennel et al. [18] (2015); Wilder et al. [19]; Kuehnert et al. [20]; and Sharma [21].

The CHNA is a systematic process involving the community to identify and analyze community health needs. The process provides a way for communities to prioritize health needs and to plan and act upon unmet community health needs.

Sharma [21] created a conceptual model for a community health assessment divided into eight steps: (1) *know thyself*, (2) *know the community*, (3) *creating a participatory infrastructure*, (4) *developing a strategic plan*, (5) *establishing feedback mechanisms*, (6) *establishing priorities*, (7) *selecting interventions* and (8) *presentation of a joint report*.

The Patient Protection and Affordable Care Act [22] has demanded that nonprofit hospitals must conduct a CHNA once every three years. The purposes are to adapt health services, implement strategies to address health priorities and improve population health.

Another tool, used in South Korea, is the Comprehensive Health and Social Needs Assessment (CHSNA) by Park et al. [23]. This validated system is characterized by user-friendly images and can be used by healthcare professionals, social workers and community residents to evaluate the reasoning underlying health and social needs, to facilitate the identification of more appropriate healthcare plans and to guide community residents to receive the best healthcare services. In detail, the assessment covers three areas: a basic health assessment, a life and activity assessment and an in-depth health assessment.

Through the literature research, another measurement method has been identified, such as the Community-Based Collaborative Action Research (CBCAR) by Van Gelderen et al. [24] and Krumwiede et al. [25]. This tool seems to facilitate community engagement and promote critical dialogue.

The Community-Based Participatory Research (CBPR) [13] is a partnership approach to research that equitably involves community members, organizations and researchers in all aspects of the research process. All partners shared expertise, decision-making and ownership. The aim of this tool is to increase the knowledge and understanding of a given phenomenon and to integrate the knowledge gained with interventions for policy or social change benefiting the community members.

The Precede–Proceed Model [26] is a cost–benefit evaluation framework proposed in 1974 by Green that could help health program planners, policy makers and other evaluators to analyze situations and design health programs efficiently. It provides a comprehensive structure for assessing health and quality of life needs, and for designing, implementing and evaluating health promotion and other public health programs to meet those needs.

The Participatory Rural Appraisal (PRA) [27] is an approach used by nongovernmental organizations (NGOs) and other agencies involved in international development that incorporates the knowledge and opinions of rural people in the planning and management of projects and programs.

Other tools, specific for certain categories of the population or patients, are used; for example, the Carer Support Needs Assessment Tool (CSNAT), used by Horseman et al. [28], Alvariza et al. [29], Ewing et al. [30] and Aoun et al. [31] is an evidence-based tool that enables the comprehensive assessment of carers’ support needs, facilitating tailored support for the family members and friends of adults with long-term, life-limiting conditions (palliative care, motor neuron disease, etc.). It comprises 14 areas of need in which carers commonly request support. Carers may use this tool to state what they need both to allow them to care for their family member or friend and to preserve their own health and well-being within the caregiving role.

The Questionnaire for Assessing Community Health Nurses’ Learning Needs [32] is destined for community health nurses.

The EASY-care [33], is a comprehensive geriatric assessments (CGA) instrument designed for assessing the physical, mental and social functioning and unmet health and social needs of older people in community settings or primary care.

The SPICE assessment tool [34], a shorter version of the Camberwell Assessment of Need for the Elderly, has been developed for routine use in primary care, focusing on five domains: Senses, Physical ability, Incontinence, Cognition, and Emotional distress (SPICE).

The University of Kansas Community Tool Box and the HRSA Compliance Manual have been used by Burns et al. [35] to conduct a needs assessment aimed at enhancing the service delivery of African-American adolescents and young adults at an urban federally qualified health center.

Finally, a modified version of the Patient Centered Medical Home Assessment (PCMH-A) has been developed by Kimble et al. [36] to assess primary care nurses’ perceptions of their practice.

Some other tools reported in literature are: the Community Health Assessment toolkit [37], Mobilizing for Action through Planning and Partnerships (MAPP) [38], State Health Improvement Planning (SHIP) Guidance and Resources [39], Community Health Assessment and Group Evaluation (CHANGE) [40], Needs Assessment, Resource Guide [41], Healthy People 2030 and MAP-IT [42].

Moreover, some data collection methods expressed in the literature are: the triangulation of data, methods and researchers [13,26], surveys [43] and public database consultation [19], focus groups [13,30,44,45,46,47], questionnaires [13,27,47,48], semi-structured interviews in person, by post or telephone [13,15,27,43,45,49,50] and, in particular, to a community’s key members [13,19] and technological tools such as visual tools [27], video clips [15] and applications for smartphones, tablets and PC [46].

Among these methods, those that deserve further exploration for their flexibility, innovativeness, effectiveness in identifying a community’s needs and for the involvement and empowerment of citizens are listed below.

− The elaboration, with the help of a local artist, of a “visual tool” [27], similar to a board game, submitted to citizens in order to identify and quantify the different needs of the population.
− The creation of a short video [15] in which some citizens are interviewed with the purpose to “give voice” to the minorities of the community. These videos have been used not only as a source of data for the assessment but also as a proposal to integrate the point of view of the community to the CHNA process through their direct participation.
− The use of applications and technological devices for community needs assessments [46].
− The planning of tools for needs assessments of people with low literacy skills [46].

Another important source of information which requires dedicated deepening, with transversal value at the international level, is the *Community Health Needs Assessment—An introductory guide for the family health nurse in Europe* [9]. It is a tool designed for services planning at the level of families, communities and populations, highlighting the importance of the nurses’ contribution in the process. It describes how the evaluation of needs can identify priorities, directing resources to address inequalities and to activate a mechanism of involvement and participation of the local population.

The first part of the tool provides practical and user-friendly guidance to nurses through some general definitions and more specific advice regarding the needs assessment, dividing the process into three sections: *profiling of the population*, *how do you find out* and *what to do with the information*. The second part is a training pack written for trainers involved in nursing education about community health needs assessments.

Lastly, ‘A Framework for Community Health Nursing Education’ is a document produced by WHO [51] representing a possible approach to analyzing the community context through a participatory process between the reference stakeholders of the territory.

An overview of the common aspects of these tools with the WHO framework is reported in Table 2.

**Table 1:** Chronological overview of the studies.

Authors and Year	Title	Setting	Study Design and Methods	Purpose	Sample and Tool Used	Main Findings	Strengths and Weaknesses
Sharma R. K. (2003) [21]	Putting the community back in community health assessment: a process and outcome approach with a review of some major issues for public health professionals.	USA. Primary and Community Care.	State of the science review. Method: Mixed method with conceptual model of a “process focused” approach.	Purpose: To present a model that orients the CHA (Community Health Assessment) process to community involvement.	Tool: Community health needs assessment (CHNA) process.	Definitions of: CHA, need assessment, community, health determinants were examined. A conceptual model for the CHA has been created, divided into eight phases.	Strengths: - The importance of population involvement and empowerment process; - The identification of a conceptual model for CHA. Weaknesses: - The study is dated, from 2003; - It is a US-generic and context-based study; - The nursing role is not analyzed.
Robertson J. F. (2004) [52]	Does advanced community/public health nursing practice have a future?	Illinois, USA. Primary Healthcare/Public Health.	State of the science review.	Purpose: To examine issues affecting the current and future state of community/public health nursing (PHN) with reference to the master’s degree level.	Sample: Nursing education system in the USA.	Some issues that influence the master’s degree in community nursing and public health have been identified, such as: - A lack of unambiguous professional and normative definitions about the role; - Bio-medicalization of the care system; - A lack of funds for training and departments of PHC; - A decrease of PHNs. Hypotheses for solutions are identified: - Update the definition of advanced nursing practice including the skills of population health management; - Institutional reforms; - Increasing the funding for training; - The master’s degree should be a necessary requirement to work in PHC management; - Encourage nurses to produce evidence and literature.	Strengths: - Although the article is not recent, there are many points in common with the Italian context, such as difficulties due to the lack of formal recognition of the professional role and limited funds; - Emphasis is placed on the role of assessments as a fundamental element of nursing practice in the community/public health and the production of evidence and literature on professional practice. Weaknesses: - It is a dated article; - The context (the study analyzes the American education and legal systems); - The assessment process is not investigated; - No specific tool for the assessment has been evaluated.
Yoshioka-Maeda, K., Murashima, S. and Asahara, K. (2006) [50]	Tacit knowledge of public health nurses in identifying community health problems and need for new services: a case study.	Tokyo, Japan. Public Health.	Qualitative study. Method: The case study method was used, especially the multiple case study design.	Purpose: To explore the tacit knowledge of public health nurses in identifying community health problems and developing relevant new projects.	Sample: Nine Japanese public health nurses (Tokyo) who had created new projects in their municipalities were selected by theoretical sampling and interviewed in 2002–2003.	All nine public health nurses used similar approaches to identify community health problems and needs to create new services, even though their experiences differed and the type of project varied. The approach consists of: identifying a person’s difficulties, recognizing people who have the same problems and clarifying the limits of existing services. Then, they managed to create a new project by examining individual health problems in the context of their community characteristics, social factors and using existing policies to support their clients. It is important to work on community health problems with interdisciplinary staff/teams to solve them.	Strengths: - The nursing role is analyzed; - Skills that public health nurses use to manage people’s health problems have emerged; - Importance is given to the assessment process of both the individual and the community, however starting first from individual cases to then get to the general population. Weaknesses: - It is not a recent study (2006); - The study was conducted in Tokyo, a very different context from the national one, and on PHNs and non-family and community nurses.
Running, A., Martin, K. and Tolle, L. W. (2007) [27]	An innovative model for conducting a participatory community health assessment	Utila, Honduras. Primary Healthcare/Public Health.	Qualitative descriptive exploratory study. Method: Semi-structured interviews and direct observation.	Purpose: To describe the perceived community health needs of the residents of Utila and to provide an example of a cross-cultural enhancement of these perceived health needs.	Sample: A convenience sample of 21 Utilan residents. The sample included 7 men, 14 women, 5 Latino, 4 Black and 12 Caucasian-mixed residents ranging in age from 20 to 81 years.	Community-based experiences are reported (Ecuador, Japan, Los Angeles and Kenya) which support the thesis that the process of assessing needs is conducted with an approach and a tool that is as specific as possible and that allows communities to identify their own healthcare needs. This approach develops trust and therapeutic education between professionals and the community. Different types of assessment are reported including questionnaires, semi-structured interviews in person, by post or telephone and the participatory rural appraisal (PRA). The assessment process is adapted to the context and a combined approach of the different methodologies may be used. Nineteen themes emerged from interviews analysis; an innovative approach was adopted with the involvement of a local artist which allowed the construction of a graphic tool (visual tool) similar to a board game to be submitted to the population in order to identify and quantify the different needs of the community itself.	Strengths: - It emphasizes the importance of a cross-cultural approach; - The study is based on Leininger’s theories of cross-cultural nursing and the process of community involvement in Hildebrandt’s model of health empowerment; - The importance of using an approach and tools for health assessments that at the same time involve the population and are specific to the community in question; - An innovative approach that analyzes the assessment process and emphasizes nursing. Weaknesses: - It is a relatively dated study (2007); - The study was conducted in a setting different from the national one (rural island of Honduras).
Li, Y., Cao, J., Lin, H., Li, D., Wang, Y. and He, J. (2009) [26]	Community health needs assessment with precede–proceed model: a mixed methods study.	Shapingba, China. Primary Healthcare.	Qualitative study. Method: Mixed method, in particular with the precede–proceed model for needs’ assessment and triangulation of data, methods and researchers.	Purpose: To understand the community’s health problems and the range of potential factors influencing risk behaviors for priority health problems.	Sample: Two communities randomly chosen in the districts of Shapingba (SPB, China) DushiGarden and Tianxingqiao; a questionnaire was submitted. Tool: Precede–proceed model for the needs assessment.	Cardiovascular disease (CVD) was identified as a priority health problem; risk factors associated with CVD included smoking, physical inactivity and unhealthy eating behaviors, particularly among low-educated male residents. Factors that negatively influence behaviors have been classified into predisposing factors (limited knowledge, beliefs and a lack of perceived needs), enabling factors (limited access to health promotion activities, unawareness of health promotion, a lack of health promotion on work and school and an absence of political relative health promotion) and reinforcing factors (culture) and, finally, limited qualified personnel in providing health promotion in the community.	Strengths: - The use of a mixed method to have the greatest possible data through the precede–proceed model and the triangulation of data, methods and researchers. Weaknesses: - It is a relatively dated study (2009); - The study was conducted in China, a very different context from the Italian one.
Akhtar-Danesh, N., Valaitis, R. K., Schofield, R., Underwood, J., Martin-Misener, R., Baumann, A. and Kolotylo, C. (2010) [32]	A Questionnaire for Assessing Community Health Nurses’ Learning Needs.	Ontario, Canada. Primary and Community Care.	Validation study. Methods: Phase I (development and pre-testing of a questionnaire on assessment training needs) and phase II (face validity testing of the questionnaire).	Purpose: To develop and evaluate a Community Health Nurse (CHN) Learning Needs Assessment Questionnaire.	Tool: Questionnaire for assessing Community Health Nurses’ learning needs.	The validity and reliability of this tool, based on standards of practice CHN (2008), is supported but must be tested in future studies. The tool can be used by CHN employers to determine staff development areas. This study also provides an example of a questionnaire development process that can be replicated by other organizations or nations to develop a reliable and valid measurement of learning needs that reflect professional standards.	Strengths: - A questionnaire was developed to measure the learning needs of CHNs; - Importance was given to the needs for professional development. Weaknesses: - It is a dated study (2010); - The study was conducted in Canada, where there is a very different healthcare system from the national one.
Krumwiede, K.A., Van Gelderen, S.A. and Krumwiede, N.K. (2014) [25]	Academic-Hospital Partnership: Conducting a Community Health Needs Assessment as a Service Learning Project.	Madelia, Minnesota, USA. Community Care.	Qualitative study. Method: Case study analysis.	Purpose: To trial the application of the Community-Based Collaborative Action Research (CBCAR) framework in nursing students while conducting a community health needs assessment and to assess the effectiveness of the CBCAR framework in providing real-world learning opportunities for enhancing baccalaureate nursing students’ public health knowledge.	Sample: Fifteen nursing students partnered with collaborative members of the Madelia Community-Based Collaborative (MCBC) group. Tool: Community-based collaborative action research.	Students developed skills in six of the eight domains of the Quad Council’s core competencies for public health nurses: 1. Analytic assessment skills. 2. Policy development/program planning skills. 3. Communication skills. 4. Cultural competency skills. 5. Community dimensions of practice skills. 6. Basic public health science skills. Community-Based Collaborative Action Research facilitates collaborative partnerships and relationships throughout the research process. Students applied what they have learned to a real community that lacks resources.	Strengths: - It emphasizes nursing students education; - It identifies the nurse as a key figure. Weaknesses: - The assessment is specific to the study context and hardly applies to the Italian context.
Kuehnert, P., Graber, J. and Stone, D. (2014) [20]	Using a Web-based tool to evaluate a collaborative community health needs assessment (CHNA).	Illinois, USA. Primary and Community Care.	Cross-sectional descriptive study. Method: The data collection was carried out with a survey (New York State Community Health Assessment Usefulness Survey).	Purpose: To describe a 2011–2012 CHNA of Kane County, Illinois.	Sample: Community leaders and members from a different set of professional backgrounds were identified with convenience sampling (N = 1913, only 262 completed the survey).	The web-based survey was defined as reliable and valid, investigated using the New York State Community Health Assessment Usefulness Survey, to measure Kane County users’ perceptions of the CHNA’s content, format and usefulness. Respondents positively evaluated the Kane CHNA assessment, although respondents who were not involved in the CHNA process were less positive than those directly involved.	Strengths: - The CHNA process is analyzed and the New York State Community Health Assessment Usefulness Survey is used for the CHNA assessment. Weaknesses: - A low survey response rate; - The study was conducted in the USA; - The nursing role is not analyzed.
Aoun, S.M., Grande, G., Howting, D., Deas, K.,Toye, C., Troeung, L., et al. (2015) [31]	The Impact of the Carer Support Needs Assessment Tool (CSNAT) in Community Palliative Care Using a Stepped Wedge Cluster Trial.	Perth, Australia. Silver Chain Hospice Care Service (SCHCS), Australia’s largest provider of home based palliative care.	Stepped-wedge cluster non-randomized trial.	Purpose: To investigate the impact of the CSNAT to identify and address support needs in end-of-life home care and on family caregiver outcomes such as strain, distress and mental and physical health; to describe implementation strategies.	Sample: Primary family caregivers of terminally ill patients (with cancer or non-cancer diagnoses) referred to Silver Chain Hospice Care. Tool: CSNAT.	The CSNAT implementation led to an improvement in caregiver strain during the caregiving period within the research context. Effective implementation of an evidence-informed tool represents a necessary step towards helping palliative care providers better assess and address caregiver needs.	Strengths: - Demonstrates the usefulness of CSNAT and identifies it as a priority for caregivers. - CSNAT was positively rated by both caregivers and nurses. Weaknesses: - Study conducted in Australia; - CSNAT is a useful assessment tool but very specific one (although it can be used by the nurse).
Craig, C., Chadborn, N., Sands, G., Tuomainen, H. and Gladman, J. (2015) [33]	Systematic review of EASY-care needs assessment for community-dwelling older people.	Primary and Community Care.	Systematic Review (SR) of the literature.	Purpose: to examine the reliability, validity and acceptability of EASY-Care and its adequacy to assess the needs of older people living in the community.	Sample: Twenty-nine papers met the inclusion criteria and underwent data extraction. Tool: The EASY-care needs assessment.	From SR the reliability tests for EASY-Care are minimal, validity tests are good and have received numerous positive approvals of acceptability in international contexts from elderly people and professionals. Finally, the tests support the use of EASY-Care for the assessment of individual needs; The data showed that among the professionals who could use EASY-Care, the majority are nurses.	Strengths: - Recent study (2015); - Systematic Review confirming the use of EASY-Care to assess the needs of older people living in the community; - The thesis is supported that the tool should be administered by the nurse. Weaknesses: - Identify one tool not yet validated in Italy but potentially useful.
Pennel, C. L., McLeroy, K. R., Burdine, J. N. and Matarrita-Cascante, D. (2015) [17]	Non-profit hospitals’ approach to community health needs assessment.	Texas, USA. Primary and Community Care.	Quantitative study (unspecified). Method: Data were obtained from multiple surveys (CHNA) conducted from 2013 to 2014.	Purpose: A better understanding of how non-profit hospitals are complying with the 2010 CHNA Patient Protection and the Affordable Care Act.	Sample: An internet search of 95 non-profit hospitals in Texas that have performed the CHNA. Tool: The CHNA process.	The main result is the wide diversity in CHNA approaches and in the quality of reports. Consultant-led CHNA processes and collaboration with local health departments have been associated with higher quality reporting. Sixteen specific criteria were identified for the evaluation of the CHNA.	Strengths: - A recent study (2015); - Sixteen specific criteria are identified to evaluate CHNAs and related bibliographic sources of reference. Weaknesses: - The study was conducted in the USA, where the practice of CHNA is different and, as reported by the authors, legislation has not yet been envisaged for specific guidelines, only generic guidelines. - The nursing role is not analyzed.
Pennel C.L., McLeroy K.R., Burdine J.N., Matarrita-Cascante D. and Wang J. (2016) [18]	Community Health Needs Assessment: Potential for Population Health Improvement.	Texas, USA. Primary and Community Care.	Mixed-method study. Methods: Two phases: a content analysis of 95 CHNAs and implementations (Texas, USA) and interviews with key informant consultants.	Purpose: To examine the population’s health promotion through planning and CHNA processes of non-profit hospitals according to the Internal Revenue Service (IRS).	Sample: A total of 95 CHNAs conducted in Texas and interviews with 16 key informants.	Although the CHNA is a great opportunity for non-profit hospital assessment and planning processes to influence the population’s health outcomes, the results of the first 3-year assessment and planning cycle (2011–2013) suggest that this is unlikely. The study offers some recommendations for improving population health, such as: clarifying the purpose of the IRS CHNA regulations, involving community stakeholders in collaborative assessment and planning, understanding the etiology of the disease, identifying and addressing broader health determinants, adopting a public health evaluation and planning model and emphasizing the improvement of population health.	Strengths: - It is a recent study (2016); - The study offers some recommendations for improving the health of the population. Weaknesses: - The role of nurses is not specified; - The study was conducted in the USA.
Wilder, V., Gagnon, M., Olatunbosun, B., Adedokun, O., Blanas, D., Arniella, G. and Maharaj-Best, A. C. (2016) [19]	Community Health Needs Assessment as a Teaching Tool in a Family Medicine Residency.	New York, USA. Primary Healthcare.	Qualitative study. Method: Primary and secondary data collected using a mixed method through public databases, surveys, focus groups and interviews with key informants.	Purpose: A description of the Community Health Needs Assessment (CHNA), as a practical way to teach research skills, community involvement and the social determinants of health.	Sample: During their one-month work in community medicine, the first-year class of 15 doctors were trained in the use of CHNAs (including directors, doctors with up to 30 years of community experience, methodologists, etc.), in Harlem, NY. Tool: CHNA process.	The study was carried out in four phases (and specific methodologies) to carry out an assessment process as complete as possible (interviews, focus groups, interviews with key informants, reviews of public data in the database and the creation of questionnaires). Among the results emerged: improving awareness of a culturally specific, feasible and accessible action for primary care. The study shows that CHNAs offer to family and community medicine an opportunity to gain a greater understanding of the issues affecting the health of patients that goes beyond just a medical examination. In addition, it is considered a useful tool for training.	Strengths: - It is a recent study (2016); - The CHNA is considered a useful tool for training within the community; - Although this study involves doctors with extensive experience in the field of primary care, postgraduates and students, the CHNA approach is used, which is considered a useful tool especially if it allows the involvement of the largest number of professions, in addition to the medical ones, such as nursing. Weaknesses: - The nursing role is not analyzed; - The study was conducted in the USA with a specific CHNA process.
Cain, C. L., Orionzi, D., O’Brien, M. and Trahan, L. (2017) [15]	The Power of Community Voices for Enhancing Community Health Needs Assessments.	Minnesota, USA. Primary and Community Care.	Quantitative study. Method: Mixed method, in particular data obtained from multiple surveys (CHNA) conducted from 2013 to 2014, integrated with semi-structured interviews with citizens (Minneapolis).	Purposes: (1) To describe a model for integrating the “voices” of community members through a qualitative approach that seeks to stimulate discussions about community needs, while also providing a new perspective on how community members think about the role of hospitals in their health. (2) Use the results of these qualitative interviews to discuss three issues that emerged.	Sample: Citizens identified among the population in Minneapolis (Minnesota) (convenience sampling) and belonging to Abbott Northwestern Hospital and prevention services. Tools: The CHNA process and specific semi-structured interviews.	Several interventions have been identified to improve the health of the local communities: community members have requested that hospitals treat culture as a health resource, not just something to be treated with “sensitivity”. They discussed how supporting community connection can encourage activities to improve physical health. Finally, they demanded health organizations to be present through real engagement with community members and taking time to listen to citizens.	Strengths: - It is a recent study (2017); - It offers an innovative way of assessment (recording of interviews); - It supports, through a qualitative study, the contribution of the population to the identification of strategies for improving health and activates a process for involving the population; - Culture is considered an essential element to be integrated into the care process. Weaknesses: - The study was conducted in the USA; - The nursing role is not analyzed.
Coats, H., Paganelli, T., Starks, H., Lindhorst, T., Starks A., Mauksch, L. and Doorenbos, A. (2017) [53]	A Community Needs Assessment for the Development of an Interprofessional Palliative Care Training Curriculum.	Seattle, Washington, USA. Palliative Care Training Center.	Cross-sectional descriptive study. Method: Mixed method.	Purpose: To describe the process and results of the community needs assessment and interprofessional palliative care educational needs in Washington state.	Sample: A total of 88 key informants who could represent the different palliative care professionals or stakeholder groups that the training program might serve (lawyer, community activist, complementary therapy—for example music and massage—physician’s assistant and psychology).	The multiple phases of the needs assessment helped to create a conceptual framework for the Palliative Care Training Center and developed an interprofessional palliative care curriculum. This curriculum will provide an interprofessional palliative care educational program. The key informant interviews also identified four central content areas for the interprofessional curriculum: 1. patient and family communication; 2. symptom management; 3. communication for care coordination; 4. organizational and cultural change.	Strengths: - The study gives importance to interdisciplinary work and made it possible to create an interdisciplinary curriculum. Weaknesses: - The study was conducted in the USA (Washington); - It is very specific for palliative care and focused only on professionals and not on the community; - The role of nurses is not highlighted; - Community assessment tools were not identified.
Evans-Agnew, R., Reyes, D., Primomo, J., Meyer, K. and Matlock-Hightower, C. (2017) [16]	Community Health Needs Assessments: Expanding the Boundaries of Nursing Education in Population Health.	Tacoma, Washington, USA. Public Health.	Case study.	Purpose: To describe how baccalaureate practicum experience within such an assessment process, involving healthcare system partners, re-affirms the importance of community and population health assessment in the development of future nursing leaders.	Sample: University students of nursing (Tacoma, USA).	Student assessments indicated an emerging appreciation for the social determinants of health, the power of partnerships and the importance of diversity. The integration of healthcare and public health system perspectives on assessments meets both public health and nursing accreditation standards and extends student leadership experiences. This integration also improves the regional capacity to improve the population’s health state. In conclusion, federal mandates for a community health needs assessment provide opportunities to advance leadership roles for nursing graduates throughout the health system and to confirm the importance of community assessments as an essential nursing competence.	Strengths: - It is a recent study (2017); - Nurses and nursing students are examined and importance is given to the community needs assessment process as a core competence of community/public health nurses (C/PHN). Weaknesses: - The study was conducted in the USA, with a cultural context different from the Italian one.
Massimi, A., De Vito, C., Brufola, I., Corsaro, A., Marzuillo, C., Migliara, G., et al. (2017) [54]	Are community-based nurse-led self-management support interventions effective in chronic patients? Results of a systematic review and meta-analysis.	Primary and Community Care.	Systematic review of the literature and meta-analysis.	Purpose: To assess the efficacy of nurse-led self-management support versus the usual care, evaluating patient outcomes in chronic care community programs.	Sample: SR on 29 papers that met the inclusion criteria. Meta-analyses on systolic (SBP) and diastolic (DBP) blood pressure reduction (10 studies, 3881 patients) and HbA1c reduction (7 studies, 2669 patients) were carried out.	The pooled mean differences were: SBP: 3.04 (95% CI −5.01Ð−1.06), DBP: 1.42 (95% CI −1.42Ð−0.49) and HbA1c: 0.15 (95% CI −0.32 ± 0.01) in favor of the experimental groups. A meta-analyses of the subgroups showed, among others, a statistically significant effect if the interventions were delivered to patients with diabetes (SBP) or CVD (DBP), if the nurses were specifically trained, if the studies had a sample size higher than 200 patients and if the allocation concealment was not clearly defined. The effects on other observer-reported outcomes (OROs) and patient-reported outcomes (PROs) as well as quality of life remain inconclusive.	Strengths: - A recent (2017) Italian meta-analysis and systematic review; - It supports the importance of primary care and community-based services both to reduce the misuse of hospitals and appropriate care; - It values the role of nurses in self-management and in the care of patients with long-term conditions; - The study shows the importance of training. Weaknesses: - It is a generic study, with no mention of assessing the needs of the community or the use of specific tools.
Alvariza, A., Holm, M., Benkel, I., Norinder, M., Ewing, G., Grande, G., Håkanson, C., Öhlen, J. and Årestedt, K. (2018) [29]	A person-centered approach in nursing: Validity and reliability of the Carer Support Needs Assessment Tool.	Sweden. Home Palliative Care.	Validation study. Method: Validation in three stages (conceptual, semantic and operational).	Purpose: To translate and evaluate the validity and reliability of the Carer Support Needs Assessment Tool (CSNAT). It was developed in the UK especially for use among family caregivers in palliative care to provide a direct and comprehensive assessment of their support needs.	Sample: Swedish family caregivers and nurses in a home palliative care setting. Tool: CSNAT.	The study adds validity to the CSNAT (UK) and also shows that it is reliable and stable for use among family caregivers in home palliative care. The CSNAT allows for a comprehensive, person-centered approach to family caregiver assessment and support, which is facilitated by professionals but guided by family caregivers. The CSNAT approach can be repeated, allowing family caregivers to express their changing needs and to support nurses when communicating with them.	Strengths: - It is a recent study (2018); - The CSNAT has been shown to have good psychometric properties of validity for assessing the caregiver needs for nursing support in home palliative care. Weaknesses: - The CSNAT is a useful assessment tool but a very specific one (although it can be used by nurses).
Akintobi, T. H., Lockamy, E., Goodin, L., Hernandez, N. D., Slocumb, T., Blumenthal, D., Braithwaite, R., Leeks, L., Rowland, M., Cotton, T. and Hoffman, L. (2018) [13]	Processes and Outcomes of a Community-Based Participatory Research-Driven Health Needs Assessment: A Tool for Moving Health Disparity Reporting to Evidence-Based Action.	Atlanta, USA. Primary Healthcare.	Quantitative study. Method: Mixed method through community-based participatory research (CBPR), semi-structured interviews, the use of questionnaires and focus groups.	Purpose: The community-based participatory research (CBPR) health needs assessment is conducted using this tool and to implement, support and research prevention strategies for the population by the Morehouse School of Medicine Prevention Research Center (MSM PRC).	Sample: A convenience sampling of citizens in the Research Partner Communities (RPC), in Atlanta, USA. Tool: Community-based participatory research (CBPR) and CHNAs.	The health priorities of the population have been identified, including: hypertension, diabetes, obesity, sexually transmitted infections, lack of social and family cohesion, limited or non-existent opportunities for physical exercise, etc. MSM PRC research and prevention initiatives have been implemented in direct response to the priorities identified through the CBPR approach and CHNAs, including: establishing a community-engaged research agenda based on data, policies, systems and approaches, environmental change, community-led grants and job creation.	Strengths: - It is a recent study (2018); - An ad-hoc survey has been created and submitted to the population to analyze health needs; - It offers a methodological starting point for conducting a study, especially the triangulation of data, methods and of researchers. Weaknesses: - It is a study that received significant funding to be conducted (USD 25,000) and that gives incentives (including non-monetary ones) to those who participated in the survey; - The study was conducted in Atlanta, with a context different from the Italian one.
Balsinha, C., Marques, M. J. and Gonçalves-Pereira, M. (2018) [34]	A brief assessment unravels unmet needs of older people in primary care: a mixed-methods evaluation of the SPICE tool in Portugal.	Lisbon, Portugal. Primary Healthcare.	Quantitative, cross-sectional study. Method: Sequential explanatory mixed-methods design and a complementary analysis of qualitative data deriving from self-reported questionnaires and individual patient interviews.	Purpose: To explore the usefulness and feasibility of the SPICE assessment tool, taking into account the perspectives of both general practitioners (GPs) and patients.	Sample: A total of 11 GPs and 10 nurses responsible for more than 17,000 patients. Tool: The SPICE assessment tool.	Unmet needs corresponded to 7% of the total needs and “emotional distress” was the most frequent. The SPICE tool helped identify undisclosed needs, it was well accepted and its importance in clinical evaluation was recognized by GPs and patients, despite concerns about time constraints.	Strengths: - It is a recent study (2018); - It investigates the needs of a part of the population that is considered more fragile in the context of primary care; - The tool (SPICE) is considered easy to use for assessing the elderly population. Weaknesses: - The study was conducted in Portugal and not on a community but on a target population (the frail elderly belonging to the primary care department).
Careyva, B. A., Hamadani, R., Friel, T. and Coyne, C. A. (2018) [46]	A Social Needs Assessment Tool for an Urban Latino Population.	Pennsylvania, USA. Primary Healthcare.	Quali-quantitative study (not specified). Methods: Mixed method with focus groups and the use of interactive programs via a PC.	Purpose: To explore priority social needs, identify recognizable “images” for those with low literacy skills and the perception of being able to assess these needs through technology such as a tablet.	Sample: Hispanic and non-Hispanic citizens of an urban community in Allentown, Pennsylvania, identified through six primary care services.	Three domains of social needs have been identified: access to care, health promotion behaviors and family responsibilities. Participants expressed different social needs with notable differences between the demographic groups. The perceptions regarding the use of an interactive computer program to assess social needs varied by age but most participants noted that a tablet was an acceptable way to share social needs, although training may be required for people over 65.	Strengths: - It is a recent study (2018); - It proposes the use of technology (tablet, app, etc.) for needs assessments; - The hypothesis of creating an ad hoc tool for the assessment of needs and it is also suitable for people with low literacy skills. Weaknesses: - The study conducted was in the USA, in particular in an Hispanic community; - The role of nurses is not analyzed; - No specific tool for the assessment has been evaluated.
Carlton, E. L. and Singh, S. R. (2018) [14]	Joint Community Health Needs Assessments as a Path for Coordinating Community-Wide Health Improvement Efforts Between Hospitals and Local Health Departments.	USA. Hospitals and Local Health Department/Primary and Community Care.	Quantitative study (unspecified). Method: The data was obtained from multiple surveys (CHNA) conducted from 2013 to 2015.	Purpose: To examine the association between the local health department’s (LHD) collaboration on a community health needs assessment (CHNA) and hospital investment in community health.	Sample: LHD (n = 439) in USA. Tool: The CHNA process.	LHDs who collaborated with hospitals on CHNAs were significantly more likely to be involved in joint implementation planning activities than those who did not. Conducting joint CHNAs can increase the coordination of efforts and community health improvement between hospitals and LHDs, and encourage hospital investment.	Strengths: - It is a recent study (2018); - It is shown that policies that allow coordination between local departments and hospitals during the CHNA have better outcomes (better community health, involvement in planning and investments). Weaknesses: - The study was conducted in the USA, different from the Italian context; - The nursing role in not analyzed.
Cho, S., Lee, H., Yoon, S., Kim, Y., Levin, P. F. and Kim, E. (2018) [47]	Community health needs assessment: a nurses’ global health project in Vietnam.	Vietnam. Primary Healthcare.	Multifaced rapid participatory appraisal, mixed method.	Purpose: To assess the health needs and suggest future interventions in Vietnam’s rural communities.	A total of 216 community residents, participated in a survey. Each commune had one focus group made up of 6–10 purposely sampled community leaders (n = 46). In total, 34 healthcare providers participated in the self-administrated survey.	Most citizens used primary care services with a high degree of satisfaction. However, there were needs to provide more comprehensive services including chronic diseases, and for healthcare providers to improve their competences.	Strengths: - It is a recent study (2018); - Nursing is considered a key profession for identifying the population needs and for reducing inequalities in health; - It is argued that nurses should generate evidence regarding practice, research and policy. Weaknesses: - The study was conducted in Vietnam.
Ewing, G., Austin, L., Jones, D. and Grande, G. (2018) [30]	Who cares for the carers at hospital discharge at the end of life? A qualitative study of current practice in discharge planning and the potential value of using The Carer Support Needs Assessment Tool (CSNAT) Approach.	England. National Health Service Trusts.	Qualitative study. Methods: Mixed method with focus groups, interviews and two workshops.	Purpose: To explore whether and how family carers are currently supported during patient discharge at the end of life; to assess the perceived benefits, acceptability and feasibility of using the CSNAT approach in the hospital setting to support carers.	Sample: Three National Health Service Trusts in England, in particular focus groups with 40 hospital and community-based practitioners and 22 carer interviews about their experiences of support during hospital discharge and views of the CSNAT approach. Two workshops brought together 14 practitioners and 5 carers. Tool: The CSNAT.	A novel intervention for hospital discharge: expanding the focus of discharge practice to include an assessment of carers’ support needs at the transition to help prevent the breakdown of care at home and patient readmission to hospital. The potential of the CSNAT approach is to facilitate conversations about the realities of caregiving at home towards the end of life, thereby eliciting carer concerns and enabling the provision of support.	Strengths: - It is a recent study (2018); - The CSNAT approach is found to be useful, as other studies have shown: - The CSNAT could be used as a tool for assessing the needs of a specific part of the community. Weaknesses: - The study was conducted in England, in a very specific setting and target population: caregivers of people who receive home care at the end of their life; - The CSNAT is a useful assessment tool but a very specific one (although it can be used by the nurse); - The nursing role is not analyzed.
Van Gelderen, S.A., Krumwiede, K.A., Krumwiede, N.K. and Fenske, C. (2018) [24]	Trialing the Community-Based Collaborative Action Research Framework: Supporting Rural Health Through a Community Health Needs Assessment	Minnesota, USA. Community Care.	Qualitative study. Method: Mixed methods (interviews, questionnaires and focus groups) following the Community-Based Collaborative Action Research (CBCAR) framework (partnership, dialogue, pattern recognition, dialogue on meaning of pattern, insight into action and reflecting on evolving patterns).	Purpose: To describe the application of the CBCAR framework to uplift rural community voices while conducting a community health needs assessment (CHNA) by formulating a partnership between a critical access hospital, public health agency, school of nursing and community members to improve the social health of this rural community.	Sample: The Madelia Community-Based Collaborative (MCBC) group. Tool: Community-based collaborative action research.	The CBCAR framework offered a triple benefit: 1. The critical access hospital was able to meet federal requirements. 2. The CBCAR provided a mechanism for improved community engagement and uplifting of community voices. 3. The process created meaningful public health education for nursing students. The CBCAR framework proved to be an effective and practical tool to meet the goals of community engagement, as identified by the Centers for Disease Control and Prevention; establish trusting partnerships; garner human and financial resources; enhance communication processes; and improve societal health outcomes.	Strengths: - It is a recent study (2018) that involves the population; - It identifies the key figure of the nurse; - The CBCAR allows a real assessment of needs to be carried out and with satisfactory results for the population examined. Weaknesses: - The assessment is specific to the study context and difficult to apply to the Italian context.
Haldane V., Chuah F.L.H., Srivastava, A., Singh, S.R., Koh, G.C.H., Seng, C.K., et al. (2019) [55]	Community participation in health services development, implementation, and evaluation: A systematic review of empowerment, health, community, and process outcomes.	Singapore. Primary and Community Care.	A systematic review of the literature. Method: A total of 49 studies and a narrative synthesis, developed according to PRISMA guidelines.	Purpose: To examine evidence on the outcomes of community participation in high- and middle-income countries.	Sample: In total, 49 studies and narrative synthesis.	Much evidence was unearthed which showed that community involvement has a positive impact on health, particularly when supported by strong organizational and community processes. This finding is in line with the idea that participatory approaches and positive outcomes, including community empowerment and health improvement, do not occur in a linear progression, but instead consist of complex processes influenced by social and cultural factors.	Strengths: - It is a recent literature review (2019); - Community involvement has a positive impact on health. Weaknesses: - The nursing role in not analyzed.
Horseman, Z., Milton, L. and Finucane, A. (2019) [28]	Barriers and facilitators to implementing the Carer Support Needs Assessment Tool (CSNAT) in a community palliative care setting.	Lothian, Scotland, UK. Community and Palliative Care.	Qualitative study. Method: Semi-structured interviews.	Purpose: To identify the barriers and facilitators for CSNAT implementation in a community specialist palliative care service.	Sample: Fourteen palliative care nurses from two community nursing teams in Lothian, Scotland. Tool: The CSNAT.	The study participants accepted the CSNAT and perceived it as useful but used it as an ‘add on’ to current practice, rather than as a new approach to carer-led assessments. The barriers to CSNAT use include carers’ self-deprecating attitudes and feeling that their own needs are much less important than those of the person they are caring for.	Strengths: - The CSNAT is a useful but very specific assessment tool, and it can be used by the nurse. Weaknesses: - The CSNAT is validated but can only be used in the specific target of caregivers of people at the end of their life.
Miller, K., Yost, B., Abbott, C., Thompson Buckland, S., Dlugi, E., Adams, Z., Rajagopalan, V., Schulman, M., Hilfrank, K. and Cohen, M. A. (2019) [48]	Health Needs Assessment of Five Pennsylvania Plain Populations	Pennsylvania, USA. Public Health.	Qualitative study (unspecified). Method: Surveys via a questionnaire administered via email.	Purpose: To understand the health needs of Plain (Amish and Mennonite) communities, to assess the differences between settlements and to measure how perceptions of modern medicine and technology can affect lifestyle.	Sample: Families were identified through random sampling and contacted by mail, in particular adult individuals (Old Order Amish and Old Order Mennonites) living in five settlements in Pennsylvania. Tool: An ad-hoc questionnaire used as an assessment tool.	The results of the health needs assessment are: a presence of differences from one settlement to another regarding whether respondents had a “regular” doctor, received preventive screening or vaccinated their children, with the more conservative groups generally lower in these and the less conservative higher. Respondents reported good physical and mental health compared to the general population. Despite their geographic and genetic isolation, the health of Plain communities in Pennsylvania is similar to that of other adults in the state.	Strengths: - It is a recent study (2019); - The importance of the assessment of minorities as it is often not possible to obtain information on these population groups through general data (at a national level); - An ad-hoc questionnaire is used as an assessment tool and administered to the population via email Weaknesses: - The study was conducted in Pennsylvania in Plain communities, not present in the Italian national context; - The nurse’s role is not highlighted.
Okura M. (2019) [49]	The Process of Structuring Community Health Needs by Public Health Nurses Through Daily Practice: A Modified Grounded Theory Study.	Japan. Primary Healthcare.	Qualitative. Method: The modified grounded theory approach (M-GTA) with semi-structured interviews and continuous comparative analysis using a qualitative study was performed.	Purpose: To clarify the process by which community health needs can be structured through public health nurses’ (PHNs) daily practice.	Sample: A total of 29 PHNs (inclusion criteria: work experience of at least 3 years).	Participants “used their five senses to understand the relationship between people’s health and life” and some key themes were identified: - Learning from the community; - Visiting communities frequently; - Giving importance to minorities; - Comparing the subjective and objective. Applying the results to continuing education systems can not only help to appropriately improve community health assessment methods, but can also help improve daily practice assessments and contribute to professional development.	Strengths: - It is an attempt to reconcile theoretical knowledge with daily practice; - It pays attention to the training process of professionals in PHC; - It is a recent study. Weaknesses: - The study was conducted in Japan, with a care setting and characteristics of nursing different from the Italian ones; - A very general study and, at the same time, specific results obtained for the setting in which the study was conducted; - The assessment process is not investigated; - No specific tool for the assessment has been evaluated.
Park, M., Choi, E. J., Jeong, M., Lee, N., Kwak, M., Lee, M., Lim, E. C., Nam, H., Kim, D., Ku, H., Yang, B. S., Na, J., Jang, J. S., Kim, J. Y. and Lee, W. (2019) [23]	ICT-Based Comprehensive Health and Social-Needs Assessment System for Supporting Person-Centered Community Care.	South Korea. Primary and Community Care.	Validation study. Method: The Delphi method.	Purpose: To develop a comprehensive system for the assessment of social and health needs (CHSNA) based on information and communications technology (ICT) and on the International Classification of Functioning, Disability and Health (ICF) aimed at improving person-centered community care for community residents, health professionals and social workers who provide health and social services in the community.	Sample: A total of 13 experts in medicine, nursing, public health and occupational therapy validated the CHSNA via the Delphi method.	A tool was created to assess the needs of the resident population in South Korea, validated by a group of experts. The tool features user-friendly screenshots and images. The assessment concerns: 1. A basic health assessment. 2. A life and activity assessment. 3. An in-depth health assessment. The developed CHSNA system can be used by healthcare professionals, social workers and community residents to assess processes underlying health and social needs, to facilitate the identification of the most appropriate health plans and to guide community residents to receive the best health services.	Strengths: - It is a recent study (2019) which uses innovative methods for community assessments; - There are many different professionals involved in the creation of the ICT system; - The International Classification of Functioning, Disability and Health (ICF) was used as a reference model. Weaknesses: - The nursing role is not specified; - The study was conducted in South Korea; - There are no specific details on the tool structure or the response of the population.
Poitras, M., Hudon, C., Godbout, I., Bujold, M., Pluye, P., Vallancourt, V. T., et al. (2019) [45]	Decisional needs assessment of patients with complex care needs in primary care.	Quebec, Canada. Primary and Community Care.	Multi-centered cross-sectional qualitative descriptive study. Method: Mixed method (interviews and focus groups in four institutions of the health and social services network of the primary and community care).	Purpose: To assess the decision-making needs of patients with complex care needs (PCCN) who frequently use health services.	Sample: A convenience sample of PCCNs who frequently use health services, health professionals and case managers (16 patients, 38 doctors, 6 case managers and 14 decision makers).	Interviews and focus groups were conducted and decision-making needs studied based on the Ottawa Decision Support Framework. Decision-making needs are numerous, varied and different from those of the general population, including 26 decision-making needs grouped into five themes. The most frequent decisions concern access to the emergency room, transfer to a nursing home and adherence to a plan or treatment. In addition, issues such as patients’ fear and distrust of healthcare professionals, differences of opinion between healthcare professionals and preconceived views of healthcare professionals about patients were identified.	Strengths: - It is a recent study (2019); - The study links many important aspects for the assessment process including the information needs of people and the needs of professionals; - It emphasizes the importance of shared-decision-making. Weaknesses: - The study was conducted in Canada; - The role of the nurse is not specified.
Burns, J.C., Teadt, S., Bradley, W.W. and Shade J.H. (2020) [35]	Enhancing Adolescent and Young Adult Health Services! A Review of the Community Needs Assessment Process in an Urban Federally Qualified Health Center.	Detroit, Michigan, USA. Primary and Specialty Care Services at an urban Federally Qualified Health Center (FQHC) organization in Detroit.	Qualitative study. Method: Semi-structured interviews were conducted among pediatric staff members (N = 11) using the community needs assessment approach specified for FQHCs.	Purpose: To conduct a needs assessment to enhance the service delivery of African-American adolescents and young adults (AYAs) at an urban FQHC organization in Detroit.	Sample: A total of 42 employees were interviewed by medical specialties and 460 patient satisfaction surveys were included to highlight the population’s health priorities, preferences regarding care, and the vital role that FQHCs play within the community. Tools: - The Health Resources and Services Administration (HRSA) Compliance Manual; - The University of Kansas Community Tool Box.	In this study, the community needs assessment process (CNA) is a useful tool to identify the community’s strengths and resources in order to address the social and healthcare needs of its members and must be culturally sensitive. In particular, FQHCs must perform a CNA every 3 years to accurately document the needs of the communities. This study made it possible to identify the priorities for the AYAs community (mental health, obesity and sexual health).	Strengths: - It is a recent study (2020); - The methodology and tools used; - It supports the importance of conducting the assessment, especially among the less represented categories within the community. Weaknesses: - It is a very specific study including only the target population of AYAs in Detroit; - The role of nurses is not analyzed.
Kimble, L.P, Phan, Q., Hillman, J.L., Blackman, J., Shore, C., Swainson, N. and Amobi, C.N. (2020) [36]	The CAPACITY Professional Development Model for Community- Based Primary Care Nurses: Needs Assessment and Curriculum Planning.	Atlanta, Georgia, USA. Community-Based Primary Care.	Qualitative study. Method: Mixed methods (an initial on-site meeting, data sources included team-developed pre- and post-assessment surveys and a literature review).	Purpose: To assess Registered Nurses’ (RN) perceptions of their practice in the areas of: engaged leadership, quality improvement strategy, continuous and team-based healing relationships, organized evidence-based care, patient-centered interactions, enhanced access and care coordination.	Sample: In total, 11 nurses from the CAPACITY project (which involves a partnership among Emory University’s Nell Hodgson Woodruff School of Nursing (NHWSN), Rollins School of Public Health Centers for Training and Technical Assistance and the FQHC, Mercy Care, Atlanta). Tool: A modified version of the Patient Centered Medical Home Assessment (PCMH-A) (Safety Net Medical Home Initiative, 2014).	The PCMH-A was developed by the MacColl Center for Healthcare Innovation at the Group Health Research Institute and Qualis Health. The complexity of the nursing practice within community-based primary care requires a robust approach to professional development to assure that the community-based primary care workforce is fully prepared to deliver high-quality, cost-effective care.	Strengths: - It is a recent study (2020); - Importance is given to the assessment process as an essential competence of the community nurse. Weaknesses: - It is a generic study conducted in the USA, a different setting than the Italian one; - The sample is only 11 nurses; - A tool for assessing the needs of the community is not identified; - The CAPACITY professional development project uses a modified version of the Patient Centered Medical Home Assessment (PCMH-A), with the aim of assessing the perception of nurses and not the needs of the community. This tool is created for individuals/patients and not for the community/group level.
Kim, S., Lee, T.W., et al. (2021) [43]	Nurses in advanced roles as a strategy for equitable access to healthcare in the WHO Western Pacific region: a mixed methods study.	WHO Western Pacific region (WPR), multi-country. Primary Care.	Qualitative study. Method: A mixed method divided into three phases: a descriptive survey on the current status of nurses in advanced roles in the Western Pacific region, followed by a Delphi survey and exploratory interviews.	Purpose: To identify the current status of nurses in advanced roles (NAR) in the WPR (e.g., functions, scope, competencies, educational standards, credentialing and regulation); to assess how NAR might be able to improve equitable access to quality healthcare and to identify the role of NAR in addressing future healthcare needs.	Sample: This multi-country study was conducted by the NAR study group (13 institutions from 8 countries), formed from a previously existing nursing and midwifery network related to WHO Collaborating Centers.	The study reported that NAR are not limited to clinical tasks within the hospital but are poised to active participation in primary healthcare, education/teaching, professional leadership, quality management and research. A three-level strategic framework to enhance the development of NAR was identified. 1. Micro-level (individual nurse/nursing group): increased opportunities for education, training, leadership/management capacity building and conducting research. 2. Organizational level: clear paths of a career ladder system and developing stronger networking systems at the regional level. 3. Macro-level (governmental): increasing the remuneration for higher-level roles, normative and policy support for NAR, vision and support from organizations/governments and conducting assessments to determine where NAR are most needed.	Strengths: - It is a recent study from 2021, focused on the development of the nursing role and on innovation. Weaknesses: - A different context from the Italian one (Western Pacific region); - There is no mention of a specific method or type of assessment despite this being considered an essential element.
Papadopoulou, C., Barrie, J., Andrew, M., Martin, L., Birt, A., Duffy, F.J.R. and Hendry, A. (2021) [44]	Perceptions, practices and educational needs of community nurses to manage frailty.	Scotland, UK. Primary and Community Care.	Exploratory qualitative study. Method: Focus groups and a thematic content analysis of data, facilitated by the NVivo© software.	Purpose: To understand nurses’ perceptions of frailty in a community setting and their needs for education about its assessment and management.	Sample: A total of 18 community nurses providing care to people living with frailty in a Scottish area covered by a health board with a wide range of experience, ranging from 2 to 20 years (district nursing team leaders, district nurses with a formal specialist practitioner qualification, community registered nurses and clinical support workers).	All participants thought that specific education on frailty was required and suggested that this should be incorporated into undergraduate and postgraduate nursing programs. They also identified barriers that caused a degree of frustration when managing frailty (constrained staffing levels, limited time with patients, challenges communicating with other services and difficulties navigating or accessing services or community assets). The participants expressed a need for frailty-specific education, particularly around assessments and training programs combining knowledge on how to identify, assess, prevent and manage frailty in practice while building confidence in dealing with complexity and enhancing communication and influencing skills for working with other professionals and agencies.	Strengths: - It is a recent study from 2021, focused on the development of the nursing role and on innovation; - There is emphasis on nurses’ education. Weaknesses: - It is a different context from the Italian one (Scotland); - There is no mention of a specific method or type of assessment despite this being considered an essential element; - The need for the assessment of nursing skills is identified, not on community needs.
van Vuuren, J. Thomas, J., Agarwal, G., MacDermott, S., Kinsman, L., O’Meara, P. and Spelten E. (2021) [56]	Reshaping healthcare delivery for elderly patients: the role of community paramedicine; a systematic review.	Primary/Community Care and Palliative care.	A systematic review of the literature.	Purpose: To identify evidence of the community paramedic role in the care delivery for elderly patients, with an additional focus on palliative care.	Sample: Ten studies, which were reported across thirteen articles.	Community paramedic programs had a positive impact on the health of patients and on the wider healthcare system. The role of a community paramedic was often a combination of four aspects: assessment, referral, education and communication. Limited evidence was available on the involvement of community paramedics in palliative and end-of-life care. Observed challenges were: a lack of additional training and the need for the proper integration and understanding of their role in the healthcare system.	Strengths: - It is a recent systematic review (from 2021) which analyzes the position of community paramedics and their contribution not only in emergency situations but also in preventive and rehabilitative contexts; - It stresses the importance of multidisciplinary work and the need to re-design the delivery of health services. Weaknesses: - Paramedics are not present in the Italian context; - The nursing role is not analyzed; - The study does not identify community assessment tools.

**Table 2:** Comparison of the tools identified through the literature review with the WHO “Community Health Needs Assessment” (2001).

Author, Year	TOOL	Profiling the Population			Deciding on Priorities for Action and Planning Public Healthcare Programmes	Implementing the Planned Activities	Evaluation of Health Outcomes	Multidisciplinary/Multisectoral Activity			Flexibility		Involving the Community
	CHNA Precede–Proceed Model of the assessment of needs Questionnaire for Assessing Community Health Nurses’ Learning Needs Community-Based Collaborative Action Research (CBCAR) Carer Support Needs Assessment Tool (CSNAT) EASY-care needs assessment Community-Based Participatory Research (CBPR) SPICE assessment tool, Comprehensive Health and Social-Needs Assessment System (CHSNA) HRSA Compliance Manual The University of Kansas Community Tool Box Different tools created for specific settings (questionnaires, semi-structured interviews, etc.)	Entire population	Population with specific diseases	Convenience sample (community leaders, socio-health professionals, etc.)				Nurses	Healthcare professionals	Other public services	Of the assessment process	In the use of the tool	
Sharma R. K. (2003) [21]	1	X			X	X	X		X	X	X		X
Robertson J. F. (2004) [52]	12							X					
Yoshioka-Maeda, et al. (2006) [50]	12				X	X		X					X
Running, A., et al. (2007) [27]	12		X		X			X	X		X	X	X
Li, Y., et al. (2009) [26]	2	X							X		X		
Akhtar-Danesh, N., et al. (2010) [32]	3							X					
Krumwiede, K.A., et al. (2014) [25]	4	X			X			X			X		
Kuehnert, P., et al. (2014) [20]	1			X					X	X			
Aoun, S.M., et al. (2015) [31]	5		X		X				X				
Craig, C., et al. (2015) [33]	6		X		X			X	X				
Pennel, C. L., et al. (2015) [17]	1	X							X	X		X	
Pennel C.L., et al. (2016) [18]	1	X		X	X				X	X			X
Wilder, V., et al. (2016) [19]	1	X		X					X				
Cain, C. L., et al. (2017) [15]	1;12	X		X	X				X			X	X
Coats, H., et al. (2017) [53]	12		X	X					X				
Evans-Agnew, R., et al. (2017) [16]	12							X					
Massimi, A., et al. (2017) [54]	12		X		X		X	X					
Alvariza, A., et al. (2018) [29]	5		X		X			X			X		X
Akintobi, T. H., et al. (2018) [13]	1;7			X	X	X			X		X	X	X
Balsinha, C., et al. (2018) [34]	8		X		X			X	X				
Careyva, B. A., et al. (2018) [46]	12		X		X				X		X		X
Carlton, E. L., and Singh, S. R. (2018) [14]	1	X							X				
Cho, S., et al. (2018) [47]	12	X						X	X				
Ewing, G., et al. (2018) [30]	5		X		X			X					
Van Gelderen, S.A., et al. (2018) [24]	4	X			X			X	X		X	X	X
Haldane V., et al. (2019) [55]	12	X			X						X		X
Horseman, Z., et al. (2019) [28]	5		X					X			X		
Miller, K., et al. (2019) [48]	12	X	X		X				X				
Okura M. (2019) [49]	12							X					
Park, M., et al. (2019) [23]	9			X					X			X	
Poitras, M., et al. (2019) [45]	12		X	X	X				X				
Burns, J.C., et al. (2020) [35]	10;11		X		X				X		X	X	
Kimble, L.P. et al. (2020) [36]	12							X			X	X	
Kim, S., et al. (2021) [43]	12							X			X		
Papadopoulou, C., et al. (2021) [44]	12							X			X		
van Vuuren, J., et al. (2021) [56]	12		X						X				

## 4. Discussion

Community health assessments are the basis to defining, implementing and evaluating the services and educational programs necessary to reach public health, through the definition of the main health problems and the factors influencing them, the identification of the community’s resources, the development potential and the involvement and empowerment of the people belonging to the community [9].

By identifying research using various community assessment tools, this review was able to find several recurring themes.

### 4.1. Education and Skills of the Family and Community Nurse

The relevance of education to improving how community health is assessed has been highlighted in numerous papers [19,24,25,44,47,49]. Education should be advanced [43,52] and specific to some professional fields, such as palliative care [53,54].

Evans-Agnew et al. [16] stated that the assessments of community health needs through academic and practical partnerships offer new opportunities for skills development, not only for professionals, but also for nursing students.

For education planning, it is necessary to determine the areas of competence development of family and community nurses through the assessment of learning needs [16,25,32,36,50].

### 4.2. Shared Decision Making and Nursing Role

The assessment process is defined as a core competence for the community/public health nurse (C/PHN) [9]. Nevertheless, among the selected studies, there is a limited number of those that refer to nurses, both as responsible for the assessment and as a processes member with other professionals [16,27,29,32,33,47,49,50,52].

In Cho et al.’s [47] work, it is argued that nurses play a key role in identifying the needs of the population and in reducing health inequalities.

Running et al. [27] consider nurses as professionals who can establish a real trust relationship with community members, the main actors in the assessment process [16,29,32,33,50].

Wilder et al. [19] offers a different point of view: the assessment process is carried out exclusively by doctors. Their work states that conducting a CHNA in a primary care training program can helps the next generation of family physicians become culturally competent and community-focused.

Yoshioka-Maeda et al. [50] found that providing support from PHNs to citizens considered as “difficult clients” was the starting point for identifying community health problems and the need for new services in their daily practice. The results showed that PHNs first took care of their “difficult clients” and, after, gradually identified the existence of community health problems. This is different from a traditional community assessment, in which the identification of community health problems is considered the first step in the development of a new service or action and is necessary to gather sufficient information to understand the community and to clarify its specific health problems.

In addition, community/public health nurses (C/PHN) during their daily practice make choices based on their responsibility and professional authority, determining if the different needs identified and/or problems may be addressed independently or in teams or, in general, with other professionals on an interdisciplinary level [47,50].

This perspective recognizes the usefulness of teamwork in assessment and planning: shared decision making (SDM) is an interpersonal and interdependent process in which the healthcare provider, the person and his or her family members relate to and influence each other, collaborating in healthcare decisions.

The SDM focuses on the evidence-based experiences of healthcare professionals and the unique attributes of the “patient” and her/his family [57]. This allows people to improve their knowledge of available options and clarify which ones are more important, taking into account your own values.

### 4.3. Community Engagement and Empowerment

The data produced by community assessments are as important as the process itself, because it allows a population’s engagement to be activated which leads to the empowerment of the individual and the community. The assessment process, therefore, depends on the underpinning methodological and theoretical orientation. Sharma [21] examined two possible types with different outcomes. The first has been described as a directive assessment, characterized by goals and subject matters defined by the professional, service delivery-focused, centralized decision-making, a focused task definition, a community as an object and with an expert practitioner that sees him/herself as having whole knowledge of the problem and whole responsibility for results. The second has been defined as a nondirective assessment where community members are involved in the decision-making process and play a vital role in defining their priority health needs and in taking action to meet them, with decentralized decision-making, open-ended task definitions, community as the subject and with a reflective practitioner that spends more time studying the problem and engaging the community in a dialogue regarding problems and their possible solutions.

Community engagement has a positive impact on health, particularly if supported by strong organizational and community processes [55].

The systematic review by Haldane et al. [55] argues that community participation is a key element of an equitable, rights-based approach to health that has been shown to be effective in optimizing the health interventions for positive public health outcomes in a wide range of health areas and on multiple levels: organizational, community and individual.

Indeed, engagement makes it possible to establish trusting partnerships, to collect human and financial resources, to enhance communication processes and to improve health outcomes [24].

A theme closely related to engagement is empowerment; in fact, the participation of community members in decisions about their health reflects the process of empowerment itself [27] and is considered, along with the establishment of trusting relationships between citizens and professionals, to be a key element of health.

Furthermore, the citizens themselves express their willingness to be actively involved by health organizations [15]. Nevertheless, CHNAs often use quantitative data, revisions of the public data in databases and rarely incorporate directly the “voices” of the local community members. Then, what emerges is only an average of the data and not the specific, actual needs of the community, leading to an increasing risk to not identify and/or to underestimate the needs of some minority groups, such as ethnic minorities [15], or to not recognize the needs at the family/individual level, keeping in too-general terms.

### 4.4. “Culturally Competent” Approach

Among the examined studies, Running et al. [27] grounds its theoretical foundations on Leininger’s theories of transcultural nursing and the process of community involvement of Hildebrandt’s model of health empowerment. Several studies show the importance of using an approach and tools for the assessment that at the same time involve the general population [15,27,54] and the specific community considered, keeping a high sensitivity to the local community and minorities’ culture [15,27,31,35,45,46,48,54].

The population itself [15] asks that social health organizations treat culture as a useful resource for health.

### 4.5. Development of Social Policies

Conducting a health needs assessment can guide policies and systems, approaches to environmental change, community-administered grants and job creation [13]. Furthermore, it improves hospital community continuity [14] and reshapes the path of care of elderly or end-of-life patients [56]. All of the above requires attention to community stakeholder involvement in collaborative assessment and planning, an understanding of the etiology of diseases, identification and intervention on the broader determinants of health, adopting a public health assessment and planning model and, finally, emphasis on improving population health [18].

### 4.6. Flexibility and Local Adaptability of Tools

The CHNA may be conducted by a variety of organizations thanks to its adaptability and the possibility to customize. Every community and hospital is different in terms of resources, demographic data, health issues, partners, history and other contextual factors that contribute to the manner in which organizations and community members work together, make decisions, identify and address problems and resources. Therefore, although the tool refers to the American context, thanks to these features, it could also be applied in other countries.

However, without more specific guidance or evaluation criteria, the usefulness, the applicability and the potential improvement of community outcomes are difficult to identify [17]. For this reason, Pennel [18] gives some recommendations to improve assessments and outcomes on a population’s health.

In addition, organizations may carry out the CHNA using different methodologies, producing results that cannot be compared effectively. In fact, many authors have demonstrated information gaps [14,17,20].

The WHO [9] suggested that for several contexts, the tools can be adapted, up to the use of different tools combined each other, in order to create one that is effective and suitable for the considered community, the social and health characteristics of the citizens and for the network of services present.

The tool and the adopted approach need to be multidisciplinary and allow community engagement and empowerment [9,15,23,46].

To the best of our knowledge, this scoping review is the first attempt to provide an overview of community assessment tools, keeping the guidance provided by the WHO as a reference.

### 4.7. Limitations

This study has some limitations. First, this article does not perform a critical assessment of the literature included. However, as a scoping review, the aim of this study was not to synthetize evidence, but to pool together elements and core concepts from a various body of knowledge. The literature review was performed until May 2021, exposing this work to a publication bias. Nevertheless, the COVID-19 pandemic may also have led to differences in perspectives among studies conducted from 2020 onward compared with those from previous years.

## 5. Conclusions

Community assessments are a core competence for nurses but their role must be better defined, both as an autonomous and a collaborative one. According to Friedman [5], nurses work with individuals, families and communities at different levels and degrees.

From the literature review and the analysis of regulatory references emerges a multi-professional approach, both in assessing the health needs of the community and in the treatment of identified needs. It means that the global assessment of the community and the definition of programs and services are carried out by a multi-professional team, with the equal participation of the community members.

When nurses work with families and communities, their goal is to guide them in the identification of problems and strengths, supporting analysis and decision-making. Community health can, in fact, be defined as the satisfaction of the collective needs of its members through the identification of problems and the management of interactions within the community [58].

The multidisciplinary approach in “individuals’ and communities’ health needs identification” must not, however, leave behind the fundamental and widespread role of every nurse involved in the everyday care of individuals, as: a source for activity data collection, which will then be aggregated; indirect community health promotion, supporting the individual health; the reinforcement of the social responsibility of each citizen, through health education.

## Data Availability

Not applicable.
